# *Pseudomonas aeruginosa* in tracheal aspirate: Colonization, infection, and recurrence

**DOI:** 10.1111/crj.13612

**Published:** 2023-04-27

**Authors:** Larissa Hermann de Souza Nunes, João Felipe Bernardi Lora, Luiz Augusto Fanhani Cracco, Janice Alexandra da Costa Manuel, June Alisson Westarb Cruz, Joao Paulo Telles, Felipe Francisco Tuon

**Affiliations:** ^1^ Laboratory of Emerging Infectious Diseases, School of Medicine Pontifícia Universidade Católica do Paraná Curitiba Paraná Brazil; ^2^ Academia BAI Luanda Angola; ^3^ Pontifícia Universidade Católica do Paraná Curitiba Paraná Brazil

**Keywords:** drug resistance, *P. aeruginosa*, risk factors, ventilator‐associated pneumonia

## Abstract

**Introduction:**

*Pseudomonas aeruginosa* respiratory infections are challenging, and the risk of recurrence is a frequent problem. The aim of this study was to investigate the risk factors associated with the presence of *P. aeruginosa* , and the risk factors related to the recurrence and death of lower airway infections in inpatients in a Brazilian hospital.

**Methods:**

Retrospective cohort with inpatients that had a sample of airways culture (tracheal aspirate or bronchoalveolar lavage) with the detection of *P. aeruginosa* . The patients with clinical criteria of infection were classified as ventilator‐associated, hospital‐acquired, or community‐acquired pneumonia. *P. aeruginosa* in respiratory samples without symptoms was considered colonization. The antimicrobial treatment adequacy and the clinical data were evaluated. Outcome variables included mortality and recurrence.

**Results:**

One hundred and fifty‐four patients were included in the study, most of them were men, and the majority (102) were considered infected. The average length of stay was superior to 30 days. Previous pulmonary disease was associated with the occurrence of colonization. Aminoglycosides were the most active drug according to susceptibility tests and were successfully used as monotherapy. Septic shock was a risk factor for death in the infected patients. The use of adequate antimicrobial therapy was associated with major survival, independent of the infection classification.

**Conclusion:**

It is possible to evaluate clinical data associated with recurrence and mortality in patients with different lung infections by *P. aeruginosa* . Aminoglycoside monotherapy is safe and effective in *P. aeruginosa* respiratory infections.

## Graphical abstract

Given the recent change challenge in the treatment of the costs of *Pseudomonas* infection, the need to understand the risk of recurrence and mortality is important. This study demonstrated, despite of intrinsic limitations of the study design, that monotherapy in a short time (<14 days) is effective, and it is possible to report the susceptibility profile of this pathogen.

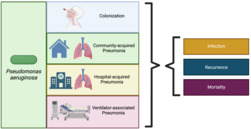

## 1 INTRODUCTION

*Pseudomonas aeruginosa* has been one of the most important etiology of hospital‐acquired infections.^1^ This pathogen is responsible for about 10%–15% of health‐associated infections (HAIs), mainly in intensive care units.^2^ One‐third of ventilator‐associated pneumonia (VAP) is caused by *P. aeruginosa* in some regions.^3^ Risk factors concerning mortality have been studied, and *P. aeruginosa* isolated in a blood culture sample is an independent risk factor for death.^4^

Considering the broad spectrum of infections and the high capacity to disseminate multidrug‐resistant strains, *P. aeruginosa* has become a public health concern. MDR *P. aeruginosa* strains already cause 13% of serious HAIs in the United States.^3^, ^5^ To highlight this scenario, *P. aeruginosa* was listed by World Health Organization (WHO), in 2018, as a critical priority for new antibiotic development.^6^

Among infections caused by *P. aeruginosa*, hospital‐acquired pneumonia (HAP) has an incidence between 5 and 20 cases for every 1000 hospital admissions, with major prevalence in elderly, postoperative, and immunocompromised patients.^7^ In these cases, the etiologic diagnosis is more difficult due to the need to collect the tracheal aspirate (TA) in a patient in spontaneous ventilation; thus, it may cause up to 37% of no agent identification.^7^

Colonization by *P. aeruginosa* is an important risk factor for VAP; therefore, the colonized patient has eight times more chances of developing VAP by *P. aeruginosa* than the uncolonized.^2^ The literature shows that oral or tracheal colonization by *P. aeruginosa* increases with the length of hospital stay, use of antibiotics, and disease severity.^1^ In patients with chronic obstructive pulmonary disease (COPD), colonization by *P. aeruginosa* was described as an independent and strong predictor factor for the increase in hospitalizations by exacerbation and increased deaths by all causes.^8^

Antimicrobial therapy against *P. aeruginosa* must be assertive because of the high morbidity and mortality associated, especially in septic patients. The correct choice of antibiotic to be administered depends on the local profile resistance, risk factors, and clinical severity. When performed assertively, it is associated with better outcomes and a lower risk of developing multi‐drug resistant agents.^9^ Considering the challenge in the treatment and recurrence of *P. aeruginosa*, the aims of this study were (1) to determine risk factors for *P. aeruginosa* lower tract respiratory infection using a control group of patients with low‐respiratory tract colonization only, and (2) to evaluate the risk factors associated with recurrence and death of lower tract respiratory infections caused by *P. aeruginosa* in a retrospective cohort of inpatients.

## 2 METHODS

This is a cross‐sectional study of patients with *P. aeruginosa* lung colonization and infections (community‐acquired pneumonia [CAP], HAP, VAP). The study included patients from January 2017 to January 2021 admitted to a Brazilian University Hospital in the South of Brazil. The hospital has 207 beds, including 30 intensive care beds, and it is a reference for trauma and surgery. This study was approved by the local ethical committee, and consent was waived because of the study design without intervention.

Inclusion criteria were patients with positive culture for *P. aeruginosa* as a single isolate in samples obtained from TA or bronchoalveolar lavage (BAL). Exclusion criteria were (1) pregnancy, (2) age lower than 18 years old, (3) diagnosis of cystic fibrosis, or (4) patients under palliative care.

Pneumonia was defined as a new and persistent pulmonary infiltrate seen on chest X‐ray in patients with a positive culture for *P. aeruginosa* (TA or BAL) and at least one of the following criteria: (1) temperature >38°C/<36.1°C; (2) leukocytes >10 000 or <4000; (3) change in sputum appearance/need for frequent aspiration. CAP, HAP, and VAP definitions followed current guidelines.^10^

CAP was defined in patients who presented the criteria mentioned above in less than 48 h of hospitalization. HAP was defined according to the healthcare‐related infection criteria: (1) infection after 48 h of hospital admission; (2) patients who were hospitalized in the last 90 days; (3) residents of nursing homes or long‐stay units, patients who received intravenous antibiotics, chemotherapy or wound care in the last 30 days and patients on hemodialysis. Lastly, VAP was defined if pneumonia occurred after 48 h of orotracheal intubation and/or mechanical ventilation. Colonization was defined as patients with TA or BAL positive for *P. aeruginosa* without criteria for pneumonia.

Treatment was defined as appropriate if one or more antibiotics initiated were active based on the susceptibility profile. Clinical cure was defined as the resolution of signs and symptoms without the need for additional antimicrobial therapy, while clinical failure was defined as persistence or progressive clinical worsening after at least 3 days of treatment. Recurrence was defined as a new TA or BAL collected based on suspicion of a new low respiratory tract infection (LRTI) after at least 4 days after the first episode of LRTI caused by *P. aeruginosa*, regardless of susceptibility to antimicrobials.

All respiratory samples were quantitative. The cut‐off of the quantitative cultures was 10^7^ cfu/mL for TA and 10^5^ cfu/mL for BAL. Matrix‐assisted laser desorption/ionization‐time of flight (MALDI‐TOF) (VITEK MS®, Biomerieux, Marcy‐l'Étoile, France) was used for bacterial identification and automated system (Walkway Microscan®, Beckman‐Coulter, Brea, CA) for antimicrobial susceptibility tests. The susceptibility interpretation was according to Clinical and Laboratory Standards Institute (CLSI).^11^

Clinical variables were evaluated, including age, gender, comorbidities, previous use of antibiotics, admission to the intensive care unit, enteral feeding, sepsis and septic shock, length of stay, and duration of mechanical ventilation. The outcomes included in this analysis were overall mortality. Risk factors for infection using a colonization group and LRTI recurrence were also evaluated.

Continuous variables were expressed mean ± standard deviation (SD) considering normalization using the Shapiro–Wilk test. Frequency was expressed as percentages. Chi‐square was used to analyze categorical variables and Student's *t*‐test for mean. A *p* < 0.05 value was considered significant. After univariate analysis, variables with *p* < 0.1 were included in a binary multivariable analysis to define independent risk factors. SPSS 25.0 (IBM, Armonk, NY) was used for statistical tests.

## 3 RESULTS

### 3.1 Infection versus colonization groups

One hundred and fifty‐four patients were included, with a median age of 55 years [36–74] and male predominance (75%) (Table 1). Most patients fulfilled the definition of infection (*n* = 102, 66%). The average length of stay was 45.3 days, and the duration of mechanical ventilation was 19.7 days. More than a third were previously hospitalized, (*n* = 58, 38%) and more than half had used antibiotics (*n* = 81, 53%). The most frequent comorbidities in our population were systemic arterial hypertension (37%), diabetes (14%), COPD (11%), heart failure (10%), bronchiectasis (6%), and chronic kidney disease (6%). Colonization was only more common in patients with abnormal pulmonary structure (i.e., COPD and/or bronchiectasis); however, no statistical difference was demonstrated.

**TABLE 1:** Univariate analysis comparing clinical data of patients with a respiratory infection and respiratory colonization by *P. aeruginosa* .

		All cases		Infection		Colonization		
		*n*	%	*n*	%	*n*	%	*p*‐value
		154	100%	102	66%	52	34%	
Male		115	75%	77	75%	38	73%	0.509
Mortality		52	34%	39	38%	13	25%	0.216
Previous admission		58	38%	41	40%	17	33%	0.101
Emergency treatment		31	20%	22	22%	9	17%	0.557
Previous use of antibiotics		81	53%	58	57%	23	44%	0.092
Chronic obstructive pulmonary disease		17	11%	7	7%	10	19%	0.205
Bronchiectasis		9	6%	3	3%	6	12%	0.533
Diabetes mellitus		22	14%	16	16%	6	12%	0.277
Chronic renal disease		9	6%	8	8%	1	2%	0.192
Neoplasm		8	5%	8	8%	0	0%	0.079
Liver disease		7	5%	5	5%	2	4%	0.655
Arterial hypertension		57	37%	43	42%	14	27%	0.023
Heart failure		15	10%	13	13%	2	4%	0.118
Admission to the intensive care unit		117	76%	86	84%	31	60%	0.097
Mechanical ventilation		116	75%	84	82%	32	62%	0.168
Enteral feeding		137	89%	96	94%	41	79%	0.268
Hemodialysis		14	9%	10	10%	4	8%	0.588
Infection classification								
	Colonization	52	34%	0	0%	52	100%	
	CAP	26	17%	26	25%	0	0%	
	HAP	37	24%	37	36%	0	0%	
	VAP	39	25%	39	38%	0	0%	
Septic shock		28	18%	28	27%	0	0%	0.008
Secondary bacteremia		5	3%	5	5%	0	0%	
Age (mean +/− SD)		55.1+/−19.3		55.9+/−19.3		53.1+/−19.2		0.428
Length of stay (days +/‐ SD)		45.3+/−44.6		47.9+/−44.7		39.3+/−44.3		0.291
Mechanical ventilation (days +/‐ SD)		19.7+/−26.7		22.3+/−30.1		12.5+/−11.7		0.080
Susceptibility profile								
	Amikacin	111	74%	83	79%	28	64%	0.041
	Aztreonam	69	70%	44	70%	25	71%	0.530
	Cefepime	97	65%	70	67%	27	61%	0.304
	Ceftazidime	95	64%	68	65%	27	61%	0.416
	Ciprofloxacin	109	73%	80	76%	29	66%	0.186
	Gentamicin	117	79%	89	85%	28	64%	0.005
	Meropenem	99	66%	70	67%	29	66%	0.537

Abbreviations: CAP, community‐acquired pneumonia; HAP, hospital‐acquired pneumonia; SD, standard deviation; VAP, ventilator‐associated pneumonia.

Among patients with infection, 74% (*n* = 76) were classified as HAIs (HAP = 37 and VAP *n* = 39). The infection group had a higher prevalence of systemic arterial hypertension compared with the colonization group (42% vs. 27%, *p* = 0.023). Other characteristics more prevalent in the infection group did not achieve statistical differences, such as higher frequency of previous antibiotics (58% vs. 23%, *p* = 0.092), more intensive care unit admissions (84% vs. 60%, *p* = 0.097), use of mechanical ventilation (82% vs. 62%, *p* = 0.168), and a higher mortality rate during the hospitalization (38% vs. 25%, *p* = 0.216).

Antimicrobial susceptibility was higher to aminoglycosides (i.e., gentamicin, 79%; amikacin, 74%). Nevertheless, in the colonization group, susceptibility to those options was lower ([gentamicin, 85% vs. 64%, *p* = 0.005], (amikacin 79% vs. 64%, *p* = 0.041]). Susceptibility to other antibiotics is described in Table 1.

### 3.2 Infection cohort: Recurrency and death groups

In univariate analysis, patients with recurrent infections presented a higher length of stay (65.5 vs. 43.8 days, *p* = 0.049) (Table 2). Additionally, septic shock was less frequent in the non‐recurrent group (29% vs. 5%, *p* = 0.0026). However, despite mortality being more frequent in patients with no recurrence, it was not significant (44% vs. 20%, *p* = 0.069). Multivariate analysis was not performed to recurrence.

**TABLE 2:** Univariate analysis comparing clinical data of patients with recurrent and non‐recurrent *P. aeruginosa* respiratory infection.

		All cases		No recurrent		Recurrent		
		*n*	%	*n*	%	*n*	%	*p*‐value
		102	100%	82	66%	20	34%	
Male		80	78%	63	77%	17	85%	0.154
Mortality		40	39%	36	44%	4	20%	0.069
Previous admission		45	44%	34	41%	11	55%	0.130
Emergency treatment		22	22%	20	24%	2	10%	0.173
Previous use of antibiotics		63	62%	49	60%	14	70%	0.166
Chronic obstructive pulmonary disease		10	10%	7	9%	3	15%	0.268
Bronchiectasis		6	6%	5	6%	1	5%	0.697
Diabetes mellitus		17	17%	13	16%	4	20%	0.380
Chronic renal disease		9	9%	7	9%	2	10%	0.520
Neoplasm		8	8%	7	9%	1	5%	0.549
Liver disease		5	5%	4	5%	1	5%	0.645
Arterial hypertension		45	44%	37	45%	9	45%	0.485
Heart failure		13	13%	12	15%	1	5%	0.263
Admission to the intensive care unit		87	85%	72	88%	15	75%	0.339
Mechanical ventilation		85	83%	70	85%	15	75%	0.463
Enteral feeding		99	97%	80	98%	19	95%	0.163
Hemodialysis		11	11%	9	11%	2	10%	0.614
Infection classification								
	CAP	26	25%	21	26%	5	25%	
	HAP	36	35%	29	35%	7	35%	
	VAP	38	37%	31	38%	7	35%	
Septic shock		25	25%	24	29%	1	5%	0.026
Secondary bacteremia		5	5%	5	6%	0	0%	0.381
Adequate therapy		81	79%	63	77%	18	90%	0.121
Age (mean +/− SD)		56.1+/−19.2		57.0+/−19.1		52.5+/−19.7		0.341
Length of stay (days +/‐ SD)		47.7+/−45.5		43.8+/−40.8		65.5+/−56.2		0.049
Days of antimicrobial therapy		9.5+/−4.4		9.6+/−4.6		9.0+/−3.3		0.583
Mechanical ventilation (days +/‐ SD)		23.4+/−31.1		24.8+/−31.1		19.9+/−36.1		0.635
Susceptiblity profile								
	Amikacin	86	58%	70	69%	16	80%	0.582
	Aztreonam	46	47%	37	36%	9	45%	0.312
	Cefepime	73	49%	58	57%	15	75%	0.307
	Ceftazidime	71	48%	57	56%	14	70%	0.410
	Ciprofloxacin	83	56%	65	64%	18	90%	0.088
	Gentamicin	92	62%	73	72%	19	95%	0.131
	Meropenem	72	48%	57	56%	15	75%	0.274

Abbreviations: CAP, community‐acquired pneumonia; HAP, hospital‐acquired pneumonia; SD, standard deviation; VAP, ventilator‐associated pneumonia.

Higher mortality in infected patients was related to septic shock (*p* < 0.001), need for ICU admission (*p* = 0.045), mechanical ventilation (*p* = 0.003), older age (*p* < 0.001), presence of heart failure (*p* = 0.028), chronic kidney disease (*p* = 0.004), and arterial hypertension (*p* = 0.007) (Table 3). Lastly, lower mortality was related to adequate antimicrobial coverage, with 86% in the survival group and 66% in the group of death (*p* = 0.025).

**TABLE 3:** The risk factor associated with death in patients with respiratory infection by *P. aeruginosa* .

		Death		Survival		
		*n* = 39	%	*n* = 63	%	*p*‐value
Male		25	64%	49	78%	0.171
Previous admission		20	51%	24	38%	0.220
Emergency treatment		9	23%	13	21%	0.807
Previous use of antibiotics		23	59%	34	54%	0.684
COPD		4	10%	5	8%	0.728
Bronchiectasis		1	3%	5	8%	0.402
Diabetes mellitus		8	21%	8	13%	0.401
Chronic renal disease		7	18%	1	2%	0.004
Neoplasm		5	13%	2	3%	0.102
Liver disease		2	5%	3	5%	>0.999
Arterial hypertension		24	62%	21	33%	0.007
Heart failure		9	23%	4	6%	0.028
Admission to the intensive care unit		35	90%	45	73%	0.045
Mechanical ventilation		36	92%	42	67%	0.003
Enteral feeding		37	95%	55	89%	0.476
Hemodialysis		6	15%	4	6%	0.175
Bacteremia		2	5%	2	4%	>0.999
Septic shock		17	44%	7	11%	<0.001
Adequate therapy		25	66%	51	86%	0.025
Age (mean +/− SD)		65 +/− 16		51 +/− 19		<0.001
Length of stay (days +/‐ SD)		49 +/− 41		41 +/− 37		0.339

Abbreviations: CAP, community‐acquired pneumonia; COPD, chronic obstructive pulmonary disease; HAP, hospital‐acquired pneumonia; SD, standard deviation; VAP, ventilator‐associated pneumonia.

In multivariate analysis (Table 4), independent factors related to mortality in infected patients were: age >56 years (HR = 2.2; [CI 95% 1.0–4.8; *p* = 0.038]), chronic kidney disease (HR = 2.52; [CI 95% 1.0–6.3; *p* = 0.05]), septic shock (HR = 2.8, [CI 95% 1.2–6.2; *p* = 0.011]), and adequate antibiotics use (HR = 0.4, [CI 95% 0.1–0.8; *p* = 0.025]).

**TABLE 4:** Multivariable analysis of risk factors for death of patients with respiratory infection by *P. aeruginosa* .

Variable	P	HR	CI 95%
Septic shock	0.011	2.8	1.2–6.2
Age >56 years	0.038	2.2	1.0–4.8
Mechanical ventilation	0.938	‐	‐
Chronic renal disease	0.05	2.52	1.0–6.3
Adequate therapy	0.025	0.4	0.1–0.8
Neoplasm	0.204	‐	‐

## 4 DISCUSSION

This study aimed to explore the related factors to the presence of *P. aeruginosa* in samples of lung secretion inpatients. This microorganism represents an important agent of infection, especially in the hospital environment where the prevalence of *P. aeruginosa* in lung infections can represent almost 20%.^12^ The great capacity to produce multidrug‐resistant strain is a factor that alert all the world and reinforces the importance of knowledge about this agent.^13^

*P. aeruginosa* was frequent in previously hospitalized patients and those who used antibiotics. This data is consistent with the knowledge about the capacity of *P. aeruginosa* to survive inside the hospital environment and the damage caused by the antimicrobial drugs to the microbiome.^14^, ^15^ The average length in the healthcare system is another factor directly related to the change in the microbiota and the occurrence of infections by *P. aeruginosa*.^16^ In this cohort, the general stay was almost 45 days, a long enough period for this event.

It is still a hard task to differentiate colonization and infection by *P. aeruginosa*, mainly in patients with risk factors for colonization, like structural lung disease.^8^ In this study, septic shock was the main condition associated with infection (28% vs. 0%). The presence or absence of other variables did not prove to be helpful in differentiating infection from colonization. However, fever, leukocytosis, or changes in imaging exams can be indicative of *P. aeruginosa* infection, and the use of quantitative cultures can also help, according to some studies.^17^

Recurrent infection patients had a longer hospital stay and milder clinical presentation, different from other trials.^18^ McCarthy et al. evaluated the recurrence of *Pseudomonas* bacteremia with higher mortality in patients with recurrence by the agent, with the presence of neoplasia as a risk factor—data not found in the present study. Another risk factor potentially influencing the risk of recurrence is the duration of treatment for the primary infection, which is also contradictory. Some studies favored a shorter treatment time (approximately 7 days), with similar mortality and cure rates, despite the literature having a longer treatment time (above 10 days) for *P. aeruginosa* as a base rate.^19^, ^20^ In the hospital of the present study, shorter treatment ranging from 5 to 7 days is favored, even with aminoglycosides.^21^, ^22^ Since 2014 we have treated *P. aeruginosa* infection with aminoglycoside monotherapy using therapeutic drug monitoring successfully, even though some guidelines recommend combination therapy despite low evidence.^21^, ^23^, ^24^ We are not in favor of monotherapy with aminoglycosides; however, we must have more scientific support, considering that these may be the only therapeutic option in some patients with carbapenemase‐producing *P. aeruginosa*.

Risk factors for mortality were advanced age, comorbidities like heart failure, chronic kidney disease, hypertension, and more severe initial clinical presentation—risk factors commonly associated with increased mortality to lung infections in general.^25^ The correct antimicrobial choice was the only modifiable factor in reducing mortality. In this study, *P. aeruginosa* presented good sensitivity to aminoglycosides—data according to a previous publication from the same hospital.^26^

It's still discussion about the introduction of one or two antibiotics in the MDR risk population, with guidelines like the Surviving Sepsis Campaign 2021 suggesting, with a weak recommendation, the introduction of two of them. In this study, this topic was not evaluated. The retrospective design was a clear limitation of this study, and the sample size was small.

In resume, *P. aeruginosa*‐infected patients had a more severe clinical presentation, septic shock, longer hospital stay, and need for mechanical ventilation than colonized patients. The introduction of adequate therapy was associated with better outcomes and lower mortality, reducing the risk of mortality by two times.

## AUTHOR CONTRIBUTIONS

**Larissa Hermann de Souza Nunes**: Manuscript draft; data review. **João Felipe Bernardi Lora**: Data review. **Luiz Augusto Fanhani Cracco**: Data review. **Janice Alexandra da Costa Manuel**: Manuscript support and final review. **June Alisson Westarb Cruz**: Data review. **Joao Paulo Telles**: Final review manuscript. **Felipe Francisco Tuon**: Statistical analysis and final review.

## CONFLICT OF INTEREST STATEMENT

The authors declare that they have no known competing financial interests or personal relationships that could have influenced the work reported in this paper.

## ETHICS STATEMENT

This study was approved by the local ethical committee (PUCPR), and consent was waived because of the study design without intervention.

## Data Availability

The data that support the findings of this study are available from the corresponding author upon reasonable request.
